# The influence of circadian rhythms and aerobic glycolysis in autism spectrum disorder

**DOI:** 10.1038/s41398-020-01086-9

**Published:** 2020-11-16

**Authors:** Alexandre Vallée, Yves Lecarpentier, Rémy Guillevin, Jean-Noël Vallée

**Affiliations:** 1 Department of Clinical Research and Innovation (DRCI), Hôpital Foch, Suresnes, France; 2 Centre de Recherche Clinique, Grand Hôpital de l’Est Francilien (GHEF), Meaux, France; 3 DACTIM-MIS, Laboratory of Mathematics and Applications (LMA), UMR CNRS 7348, University of Poitiers, Poitiers, France; 4 CHU Amiens Picardie, University of Picardie Jules Verne (UPJV), Amiens, France

**Keywords:** Molecular neuroscience, Autism spectrum disorders

## Abstract

Intellectual abilities and their clinical presentations are extremely heterogeneous in autism spectrum disorder (ASD). The main causes of ASD remain unclear. ASD is frequently associated with sleep disorders. Biologic rhythms are complex systems interacting with the environment and controlling several physiological pathways, including brain development and behavioral processes. Recent findings have shown that the deregulation of the core clock neurodevelopmental signaling is correlated with ASD clinical presentation. One of the main pathways involved in developmental cognitive disorders is the canonical WNT/β-catenin pathway. Circadian clocks have a main role in some tissues by driving circadian expression of genes involved in physiologic and metabolic functions. In ASD, the increase of the canonical WNT/β-catenin pathway is enhancing by the dysregulation of circadian rhythms. ASD progression is associated with a major metabolic reprogramming, initiated by aberrant WNT/β-catenin pathway, the aerobic glycolysis. This review focuses on the interest of circadian rhythms dysregulation in metabolic reprogramming in ASD through the aberrant upregulation of the canonical WNT/β-catenin pathway.

## Introduction

Intellectual abilities and their clinical presentations are extremely heterogeneous in autism spectrum disorder (ASD). ASD includes Asperger, autism and pervasive developmental disorder-not otherwise specified (PDD-NOS). ASD is characterized by numerous and complex etiologies, including inflammation, metabolic, environmental or genetic determinants. Nevertheless, the main causes of ASD remain unclear. ASD is diagnosed within the first 3 years of life. Social interaction impairment, repetitive and restrictive behaviors or stereotypic patterns of behaviors characterize the different symptoms of ASD^1^. Early diagnosis is major issue for better prognosis and therapeutic care^2,3^.

ASD is frequently associated with sleep disorders^4^. Brain structures and functions development are considered as dynamic processes, dependent on genetic and social/physical environmental cues, involving homeostasis processes at each time, and maturation of new functions in time. Biologic rhythms are complex systems interacting with the environment and controlling several physiological pathways, including brain development^5^ and behavioral processes^6^.

Recent studies have shown that the deregulation of the core neurodevelopmental signaling is correlated with ASD clinical presentation. One of the main pathways implicated in developmental cognitive disorders is the canonical WNT/β-catenin pathway^7^. Several genetic mutations observed in ASD are linked with the dysregulation of the WNT/β-catenin pathway through interactions between chromodomain helicase DNA binding protein 8 (CHD8) and CTNNB1 (β-catenin)^8^. In many ASD findings, the WNT/β-catenin pathway is increased^7,9–11^.

Circadian clocks have a main role in some tissues by driving circadian expression of genes involved in physiologic and metabolic functions^12^. One of the key integrator of these complex mechanisms is the canonical WNT/β-catenin pathway^13,14^. ASD progression is associated with a major metabolic reprogramming, initiated by aberrant WNT/β-catenin pathway, the aerobic glycolysis^11^. In parallel, the dysregulation of circadian rhythms (CRs) upregulates the WNT/β-catenin pathway^15^, which in turn participates to the ASD initiation. This review focuses on the interest of CRs dysregulation in metabolic reprogramming in ASD through the aberrant upregulation of the canonical WNT/β-catenin pathway.

## Circadian rhythms

CRs are major biological phenomena found in all universal processes. Their endogenous characteristic is an innate oscillation associated with a period of over 1 day. All the studied organisms show this oscillatory process. Numerous cell functions present temporal variations driven by these oscillatory and circadian ways, including gene expression, metabolic reprogramming, and molecular and cellular pathways. Different integration levels allow the study of the CRs, as endocrinal, physiological, neuronal cell behaviors. Although the coordination and the modulation of CRs are organized by specific pacemaker structures, the primary circadian oscillations are controlled at the cell level. These oscillations are determined by numerous clock genes^16^. The control of the circadian clock is based on an intracellular temporal tracking system that allows anterior organisms to change direction and thus adapt their behavior and the physiology of their life span^17^. It is well known that in many animals species, circadian clock is formed by a specific set of transcription factors which constitutes its molecular architecture. These determinants are both used in positive and negative feedback, which are modulated through a cell-autonomous manner^18^.

Endogenous oscillations generate a freewheeling period, which is close to 24 h, to maintain for the organism constant ambient conditions. These oscillators, at the molecular level, are based on the products of clock regulator genes organized in a transcriptional feedback loop. Circadian oscillations are the product of these post-transcriptional modifications of proteins^19^. A complex loop operates with clock gene transcriptional activators and in turn clock genes with a negative feedback role inhibiting their expression by disrupting the activity of their activators^20^. Several input pathways involve environmental information which interacts with the different compounds of the oscillators. The oscillators are synchronized with the 24 h solar day. The input pathways generate a day-time to transpose it by the oscillators to the output pathways. These output pathways control and regulate the expression of circadian clock genes to generate the rhythmicity. Moreover, the output pathways are predicted to be rhythmic and then controlled by the clock gene transcription factors. These compounds, in turn, regulate downstream the circadian clock genes in a time-of-day-specific manner^21^. This system can synchronize with its environmental time by its internal clock. To respect the environment, the input pathways are vital to maintain this timing for oscillators. A the process named entrainment, the input pathways can reset the activity of the oscillators to stay in a conform 24-h period of the environment^21^. Environmental cues can be detected by input pathways which in turn can modulate many mechanisms to control the activity or level of compounds of oscillators to keep a correct time-of-day expression. This phenomenon is observed in several environmental cues, including nutrition, social interactions and temperature^22,23^. Furthermore, the clock allows a strategy named gating to restrict responses to environmental cues at some day times. Diurnal mammals are insensitive to a light pulse during the day. Nevertheless, during the night, a light pulse can advance or delay the clock to synchronize diurnal mammals with the environment^18^. Environmental signals can interact with molecular oscillators in some cells in complex multicellular organisms. In unicellular organisms, each cell is modulated by oscillators in response to light^24^. However, in multicellular organisms, only a part of the cells has sensory capabilities leading to clock oscillators. The oscillators, and thus the overall rhythmicity of organisms, are concentrated into compounds including a master pacemaker and peripheral oscillators^25^. Face to these sensory inputs, the organism presents some nervous systems which possess environmental cue abilities as a central oscillators or pacemakers rather than to individual cells. In humans, the sensory clock inputs are localized in the brain, where signals from the master pacemaker leads to oscillators in some tissues of the organism.

Nonvisual retinal ganglion cells receive and perceive the light, and transmit this information to the master pacemaker (localized in the hypothalamus) through neural connections. The central pacemaker synchronizes oscillators to the other tissues by using circadian input pathways from the nervous system to peripheral cell systems. Moreover, to maintain the entrainment of these peripheral oscillators by the environment, this central system ensures that cellular oscillations within tissues are properly in phase to provide resonance between individual cellular rhythms^6^. Melatonin operates as a major synchronizer in humans and provides temporal feedback to oscillators within the nervous system to control the circadian phase and the stability of the rhythm^26^. In humans, as in other mammals, melatonin is considered as the main influencer of CRs through its action on receptors in the nervous system^27^.

The sleep–wake pattern is controlled by both CRs and homeostasis. Sleep pressure has been enhanced during the phase of waking and then decreases during the phase of sleeping. The sleep–wake pattern is controlled by the cycle of light–darkness^28^. Through a feedback, sleep–wake pattern can also control the CRs. For many studies, this pattern can be defined as an interface between environmental information (social, mood and cognition) and CRs^29^. Moreover, these two patters are influenced by melatonin^4^. Melatonin level is modulated by the light–dark exposure. Melatonin is associated with sleep initiation. Mutations in Clock, Bmal1, Cry1, Cry2 genes lead to the initiation of some alterations in sleep time and sleep fragmentation^30,31^.

### Circadian clock

**Fig. 1:**
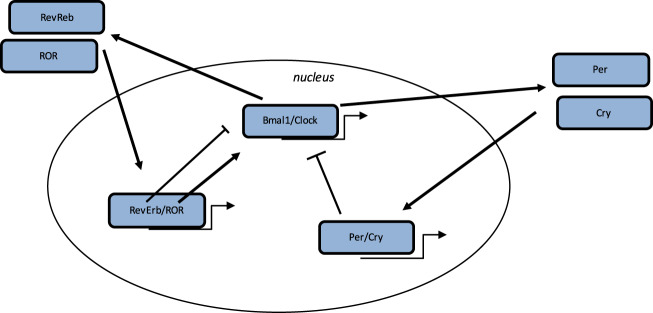
Circadian clock genes. The clock process is a stimulatory circle, involving the Bmal1/Clock heterodimer which activates the transcription of Per and Cry genes, and the inhibitory feedback circle with the Per/Cry heterodimer which translocates to the nucleus and which represses the transcription of the Clock and Bmal1 genes. An additional circle implicates the RORs and Rev-Erbs factors with a positive feedback by RORs and a negative feedback by Rev-Erbs.

Some biological mechanisms in humans are controlled by the circadian “clock” (circadian locomotors output cycles kaput) (Fig. 1). The circadian clock is localized in the hypothalamic suprachiasmatic nucleus (SCN). CRs are endogenous and entrainable free-running periods that last ~24 h. Several transcription factors can control and modulate CRs. These factors are named circadian locomotor output cycles kaput (Clock), brain and muscle aryl-hydrocarbon receptor nuclear translocator-like 1 (Bmal1), Period 1 (Per1), Period 2 (Per2), Period 3 (Per3) and Cryptochrome (Cry1 and Cry2)^32,33^. They are modulated by positive and negative self-loop-regulation mediated by CRs^18,34^. Clock and Bmal1 heterodimerize and lead to the transcription of Per1, Per2, Cry1 and Cry2 (ref. ^35^). The Per/Cry heterodimer inhibits its activation by a negative feedback. It translocates back to the nucleus to directly downregulate the Clock/Bmal1 complex and then inhibits its own transcription^35^. The Clock/Bmal1 heterodimer activates the transcription of retinoic acid-related orphan nuclear receptors, Rev-Erbs and retinoid-related orphan receptors (RORs). Through a positive feedback loop, RORs activate the transcription of Bmal1, whereas through a negative feedback loop, Rev-Erbs downregulate their transcription^35^.

## Circadian clocks in ASD

**Fig. 2:**
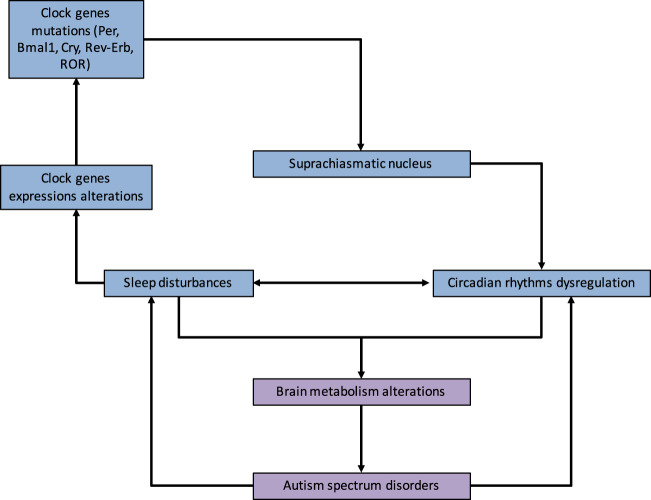
Circadian rhythms and autism spectrum disorder. Relationship between ASD, circadian rhythms and sleep disturbance. Alterations in clock genes and melatonin pathway contribute to the dysregulation of circadian sleep rhythmicity. Circadian rhythms deregulation leads to brain metabolism alterations contributing to ASD. In a negative feedback, ASD symptomatology reinforces circadian rhythms and sleep disturbances creating a self-reinforcing circle.

Sleep disorders are frequently associated with ASD (Fig. 2)^36^. Patients suffering from ASD are more correlated with falling asleep anxiety, sleep disorders and CRs disturbance^36^. Moreover, the prevalence of sleep disturbance is associated with cognitive impairments^36–38^. In parallel, melatonin levels have been dysregulated in ASD patients^4^. Melatonin diurnal levels are decreased as well as melatonin nocturnal levels, showing a global decrease in melatonin production in ASD children and a dysregulation of day–night rhythm^39–42^. Deregulation in the production of melatonin in ASD patients has been observed due to HIOMT (hydroxiindole O-methyltransferase) deficiency^43^. Recent studies have shown a potential interest in melatonin therapy in the treatment of sleep onset disorder for ASD patients^44^. Significant allelic correlation has been observed for clock genes, including Per1, and other nucleotids^45–47^. Genes involved in ASD pathogenesis are part of pathway networks enhanced in synapse formation, including Neuroxin-Neuroligin genes and in the alteration of the balance excitation-inhibition^48^. Parvalbumin expressing interneurons play a main role in ASD initiation. The knockout of Parvalbumin is associated with core symptoms of ASD patients^49^. CRs dysregulation may downregulate the maturation of Parvalbumin cells and then the timing of critical period of plasticity^50^. The dysregulation of CRs could impact the temporal organization of brain maturation and could have a cascade effect on several brain functions. Negative environmental conditions (sleep deprivation, stress…) could impact the CRs and thus redox homeostasis and transcriptional control of Parvalbumin genes involved in synapse formation and maturation of brain functions.

## Melatonin and ASD

Melatonin plays a main role in neurodevelopment^51^. Disturbances in sleep–wake rhythm in ASD could be due to the dysregulation of melatonin^52^. Sleep latency and nocturnal and early awakenings have been reported in ASD^41^. In ASD, the nocturnal secretion of melatonin is low^53^. Intellectual disabilities are associated with melatonin abnormalities^54^. However, in the Down syndrome, the production of melatonin is normal whereas melatonin production is increased in the Fragile X patients^55^. Several therapeutic studies have focused on the interest of melatonin strategy in ASD^56^. Melatonin use in ASD children is associated with improvement of communication^57^, stereotyped behaviors^58^, anxiety^59^ and social withdrawal^58^. Nevertheless, melatonin is influenced by age and pubertal stage^60^, and in these studies the autistic behavioral impairment was generally enough described^57^.

## Aerobic glycolysis

In mammalian cells, glucose is the main source of energy. All tissues need ATP to function normally. Cells produce ATP by a careful drop in oxidation state from energy-rich molecules like glucose, through the cell respiration process, down to product CO_2_ at the end. This process occurs in an aerobic or anaerobic manner, depending on whether oxygen is available. Glycolysis is the first step in glucose metabolism signaling and occurs in the cytosol of all cells. The presence of O_2_ is important because of oxidation of glucose under aerobic conditions results in ~32 molecules of ATP per molecule of glucose. Under anaerobic conditions, only two molecules of ATP can be produced. Aerobic glycolysis can occur in these two stages. The first occurs in the cytosol and involves the conversion of glucose to pyruvate resulting in NADH production and generating two molecules of ATP. In normal conditions, when oxygen is available, the energy contained in NADH is further released via re-oxidization of the mitochondrial chain and leads to the release of 30 molecules of ATP per molecule of glucose. Pyruvate is reduced to lactate, instead of re-oxidized, under aerobic glycolysis^61^. Thus, glucose is metabolized in order to produce ATP, by a cytosolic glycolysis and oxygen-dependent mitochondrial physiological mechanism. Glucose entry into the tricarboxylic acid (TCA) cycle is modulated by pyruvate dehydrogenase complex (PDH)^62^. Pyruvate is oxidized to acetyl-coA in mitochondria by the PDH. Acetyl-coA translocates to the TCA cycle for oxidation. Under aerobic glycolysis, pyruvate is converted into lactate in the cytosol by lactate dehydrogenase A (LDH-A). Moreover, aerobic glycolysis is caused by the involvement of hexokinase 2 (HK2) instead of HK1 and pyruvate kinase M2 (PKM2) instead of PKM1 (ref. ^63^). This phenomenon is called aerobic glycolysis or the Warburg effect.

## Aerobic glycolysis in ASD

Few studies have investigated the potential impact of aerobic glycolysis in ASD, and thus the expression of the glycolytic enzymes (Fig. 2)^11^. However, numerous findings have highlighted increased lactate levels in ASD patients^64–71^.

Pyruvate production is stimulated^65,67^, but with an increased ratio of lactate-to-pyruvate^65,68^ showing a high glucose metabolism and LDH-A activity in ASD patients. Moreover, LDH-A expression has been increased in ASD patients^71^. A recent study has presented a decrease level of pH correlated with high levels of lactate in ASD patients^72^. These observations could suggest an increase of aerobic glycolysis in ASD since the dysregulation of this balance has been proposed as a candidate cause of ASD^73^.

## CRs and aerobic glycolysis

Few studies have focused on the relationship between CRs and aerobic glycolysis (Fig. 2). Nevertheless, this relation could be mainly interesting in the development of tumors^74^. In the same way, melatonin expression and modulation by CRs in cancers is associated with the disruption of the aerobic glycolysis^75–77^. Thermodynamic and energy reprogramming highlight this relation in fibrosis^78^, neurodegenerative diseases^79,80^ and cancers^81^. The importance of 24-h fluctuations in the aerobic glycolysis and the availability of nicotinamide adenine dinucleotide phosphate hydrogen (NADPH) in cancer has been shown through the consideration of the redox influence on NADPH^82^.

## The canonical WNT/β-catenin pathway

**Fig. 3:**
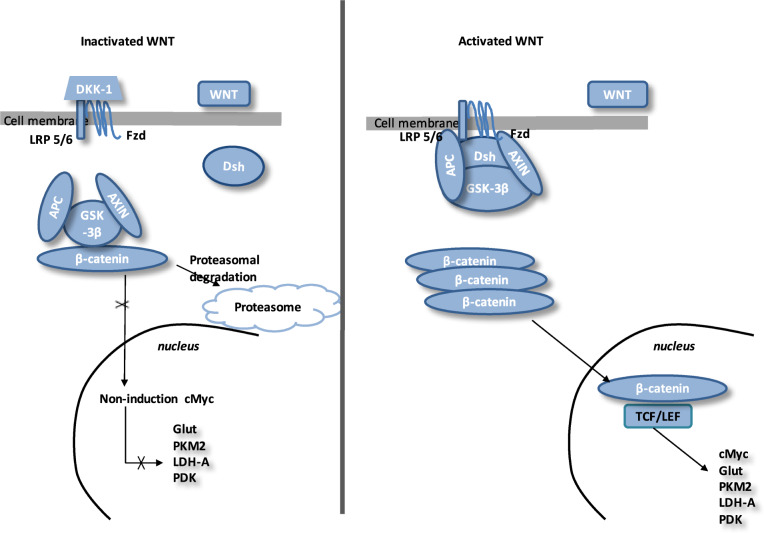
The canonical WNT/β-catenin pathway. Inactivated WNT: Under physiologic circumstances, the cytoplasmic β-catenin is linked to its destruction complex, consisting of APC, AXIN and GSK-3β. β-catenin is phosphorylated by GSK-3β. Thus, phosphorylated β-catenin is destroyed in the proteasome. Then, the cytoplasmic level of β-catenin is kept low in the non-presence of WNT ligands. If β-catenin is not accumulated in the nucleus, the TCF/LEF complex does not stimulate the target genes. DKK1 inhibits the WNT/β-catenin pathway through binding to WNT ligands or LRP5/6. Activated WNT: When WNT ligands activate both FZD and LRP5/6, DSH is stimulated and phosphorylated by FZD. Phosphorylated DSH in turn activates AXIN, which comes off the β-catenin destruction complex. Thus, β-catenin escapes from phosphorylation and then accumulates in the cytoplasm. The accumulated cytosolic β-catenin moves into the nucleus, where it interacts with TCF/LEF and stimulates the transcription of target genes.

The Wingless/Int (WNT) pathway is a family of secreted lipid-modified glycoproteins (Fig. 3)^83^. Several nervous molecular mechanisms are modulated by the WNT/β-catenin pathway, including development of synapses in the central nervous system^84,85^, synaptogenesis^86,87^ and control of synaptic formation^84,88^. Numerous pathophysiologic signalings are mediated by the dysregulation of this pathway, including cancers^89,90^, fibrosis^91^, neurodegenerative diseases^80^ and angiogenesis^13,92^. Stimulation of β-catenin signaling needs the presence of the complex LRP5 /LRP6^93^. LRP5 has a main role while LRP6 presents a minor role in the retinal vascularization^94,95^. Disheveled (Dsh) forms a complex with Axin, and this prevents the phosphorylation of β-catenin by glycogen synthase kinase-3β (GSK-3β). Then, β-catenin accumulation in the cytosol is observed and translocates to the nucleus to bind T-cell factor/lymphoid enhancer factor (TCF/LEF) co-transcription factors. This nuclear binding allows the transcription of WNT-responsive genes, such as cyclin D1, c-Myc, PDK1, MCT-1 (refs. ^96,97^).

WNT ligands’ absence is associated with cytosolic β-catenin phosphorylation by GSK-3β.

A destruction complex is composed by tumor suppressor adenomatous polyposis coli (APC), Axin, GSK-3β and β-catenin. Then, phosphorylated β-catenin is destroyed in the proteasome. WNT inhibitors, including DKKs and SFRPs, control the WNT/β-catenin pathway by preventing its ligand–receptor interactions^98^.

GSK-3β, a neuron-specific intracellular serin-threonin kinase, is the major inhibitor of the WNT pathway^99^. GSK-3β regulates numerous pathophysiological pathways (cell membrane signaling, neuronal polarity and inflammation)^100–102^. GSK-3β downregulates β-catenin cytosolic accumulation and then its nuclear translocation^100^. GSK-3β diminishes β-catenin, mTOR pathway, HIF-1α and VEGF expression^103^.

## The canonical WNT/β-catenin pathway in ASD

Some studies have observed the potential role of over-increased WNT/β-catenin pathway in ASD patients^104–106^. Furthermore, many gene compounds are associated with the development of ASD, including WNT2 ligand^107^, the WNT target MET (hepatocyte growth factor receptor)^108,109^, CHD8 (chromo-helicase domain protein 8) and DYRK1 (refs. ^110–112^). Other components of the WNT pathway have been involved in ASD pathogenesis, including WNT1 (ref. ^113^), WNT2 (ref. ^107^), WNT3 (ref. ^114^), WNT7A^115^, APC^116–118^, β-catenin^8,119^, TCF4 (refs. ^120,121^) and TCF7 (ref. ^122^). The CTNNB1 gene, a β-catenin encoder, is a major controller of the WNT pathway. Its expression is associated with sporadic ASD. Alterations of CTNNB1 correspond to intellectual disability and thus, ASD^8^. In mice, the expression of CTNNB1 expression is correlated with brain development^123^. Knockout of CTNNB1 in the Parvalbumin interneurons leads to the impairment of social interactions and enhancement of repetitive behaviors.

Phosphatase and tensin homolog protein (PTEN) inhibition is associated with the increase of the WNT pathway and high risk of ASD development^124–126^. In Purkinje cells, PTEN inhibition impairs social relation, behavior and deficits in motor learning^127,128^. PTEN and β-catenin control each other to normal brain growth and development^129^.

## CRs and WNT/β-catenin pathway

RORs are upstream effectors of the WNT/β-catenin pathway^130^. By this interaction, circadian genes can modulate the cell cycle progression^131^. A Bmal1 knockdown can downregulate the WNT/β-catenin pathway^132^. In wild-type mice, the levels of WNT-related genes are higher than those observed in Bmal1 knockdown mice^133,134^. The proliferation and progression of cell cycle are controlled by Bmal1 by activating the WNT/β-catenin pathway^135^. Bmal1 involves the β-catenin transcription, diminishes the β-catenin degradation and then inhibits GSK-3β activity^136^. In the intestinal mucosa of ApcMin/+ mice, the degradation of Per2 leads to β-catenin increase by circadian disruption^137^.

In normal conditions, the core circadian genes operate in accurate feedback loops and keep the molecular clockworks in the SCN. They allow the control of peripheral clocks^18,34^. Per1 and Per2 maintain cell CRs and modulate cell-related gene activity, such as c-Myc, so as to sustain the physiologic cell cycle^138,139^.

## Aerobic glycolysis and WNT/β-catenin pathway

**Fig. 4:**
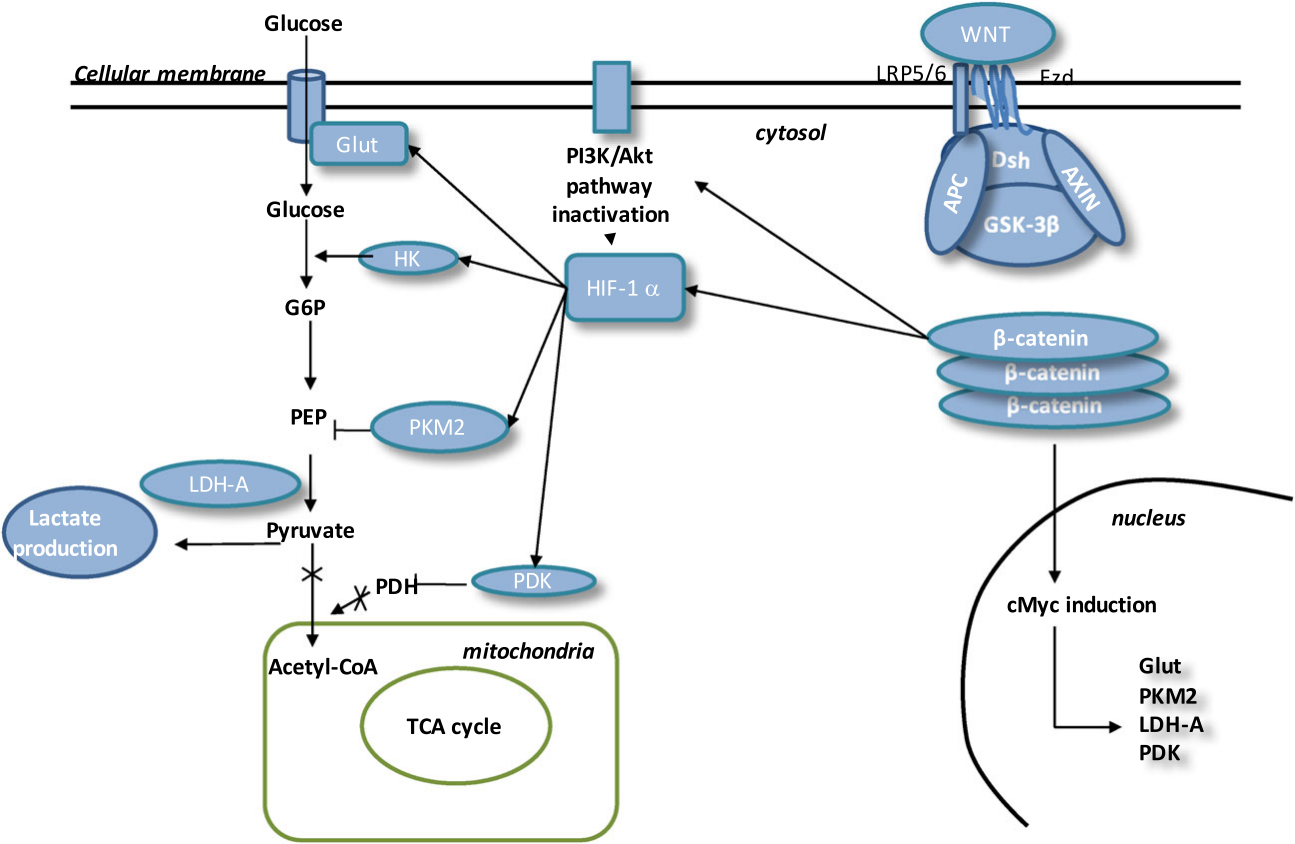
Interactions between WNT pathway and energy metabolism in ASD. In ASD, the WNT pathway is activated. In the presence of WNT ligands, cytosolic β-catenin is accumulated in cytosol and GSK-3β is inhibited. APC and Axin combine with GSK-3β and DSH to form a complex with LRP 5/6 and FZD. β-catenin translocates to the nucleus and binds to TCF/LEF co-transcription factor. WNT target genes, such as cMyc, are activated. β-catenin accumulation increases the level of PI3K/Akt pathway and results in the activation of HIF-1α. Activated HIF-1α stimulates Glut, HK, PKM2, LDH-A and PDK1. Activation of HIF-1α involves PKM2 translocation to the nucleus. PKM2 activates PEP cascade and the formation of pyruvate. PKM2 binds to β-catenin and induces cMyc-mediated expression of glycolytic enzymes (Glut, LDH-A, PDK1). Activation of Glut and HK involves glucose hyper-metabolism with increase in glucose transport and phosphorylation rates. PDK1 inhibits PDH to downregulate the pyruvate entrance into mitochondria. Lactate production is activated by LDH-A. This is aerobic glycolysis.

Some reports have highlighted that the WNT/β-catenin pathway is closely associated and a main effector of the aerobic glycolysis (Fig. 4)^11,78,140–142^. The PI3K/Akt pathway stimulates the glucose metabolism to enhance protein and lipid synthesis^143^. Moreover, PI3K/Akt pathway increases the glucose metabolism to protect cells against reactive oxygen species (ROS) stress induced by activated HIF-1α and decreasing the glucose entry into the TCA cycle^144^. HIF-1α stimulates pyruvate dehydrogenase kinase (PDK) to phosphorylate PDH and inactivates it, leading to cytosolic pyruvate being shunted into lactate by LDH-A^145^. HIF-1α is transcriptionally activated by PI3K/Akt/mTOR pathway through 4E-BP1 and STAT3 (refs. ^146–151^).

Numerous studies have observed that the WNT/β-catenin pathway can downregulate the pyruvate oxidation in the TCA cycle^140,152^. WNT/β-catenin pathway, by activating both the PI3K/Akt/mTOR pathway and HIF-1α, can lead to aerobic glycolysis^140,152,153^. PI3K/Akt pathway can also control the β-catenin accumulation and then the expression of the downstream genes^154^. c-Myc directly stimulates the HIF-1α^155^, PDK and lactate transporter (MCT-1) expressions^152^. The stimulation of HIF-1α leads to the overexpression of glucose transporters (Glut), hexokinase (HK), pyruvate kinase (PK), PDK1 and LDH-A^156–159^.

## Conclusion

Changes in energy metabolism are modulated by abnormal CRs in ASD patients. In ASD, the canonical WNT/β-catenin pathway is increased. Energy behaviors of metabolic enzymes in ASD are modified by this upregulation of the WNT/β-catenin pathway leading to the enhancement of aerobic glycolysis and thus the production of lactate. This explains the glucose hyper-metabolism observed in ASD. WNT pathway is driven by the CRs and operates under a circadian regime evolving to changes in energy metabolism. CRs directly contribute to the regulation of the molecular pathways WNT/β-catenin pathway involved in the reprogramming of cellular energy metabolism enabling ASD.
